# Metagenomic Nanopore Sequencing of Influenza Virus Direct from Clinical Respiratory Samples

**DOI:** 10.1128/JCM.00963-19

**Published:** 2019-12-23

**Authors:** Kuiama Lewandowski, Yifei Xu, Steven T. Pullan, Sheila F. Lumley, Dona Foster, Nicholas Sanderson, Alison Vaughan, Marcus Morgan, Nicole Bright, James Kavanagh, Richard Vipond, Miles Carroll, Anthony C. Marriott, Karen E. Gooch, Monique Andersson, Katie Jeffery, Timothy E. A. Peto, Derrick W. Crook, A. Sarah Walker, Philippa C. Matthews

**Affiliations:** a Public Health England, National infection Service, Porton Down, Salisbury, United Kingdom; b Nuffield Department of Medicine, University of Oxford, John Radcliffe Hospital, Headington, Oxford, United Kingdom; c Department of Infectious Diseases and Microbiology, Oxford University Hospitals NHS Foundation Trust, John Radcliffe Hospital, Headington, Oxford, United Kingdom; d Peter Medawar Building for Pathogen Research, Nuffield Department of Medicine, University of Oxford, Oxford, United Kingdom; e Oxford NIHR BRC, John Radcliffe Hospital, Headington, Oxford, United Kingdom; University Hospital Münster

**Keywords:** influenza, Nanopore, metagenomic, diagnosis, epidemiology, sequencing, DNA sequencing, diagnostics, metagenomics, molecular epidemiology

## Abstract

Influenza is a major global public health threat as a result of its highly pathogenic variants, large zoonotic reservoir, and pandemic potential. Metagenomic viral sequencing offers the potential for a diagnostic test for influenza virus which also provides insights on transmission, evolution, and drug resistance and simultaneously detects other viruses. We therefore set out to apply the Oxford Nanopore Technologies sequencing method to metagenomic sequencing of respiratory samples. We generated influenza virus reads down to a limit of detection of 10^2^ to 10^3^ genome copies/ml in pooled samples, observing a strong relationship between the viral titer and the proportion of influenza virus reads (*P* = 4.7 × 10^−5^). Applying our methods to clinical throat swabs, we generated influenza virus reads for 27/27 samples with mid-to-high viral titers (cycle threshold [*C_T_*] values, <30) and 6/13 samples with low viral titers (*C_T_* values, 30 to 40). No false-positive reads were generated from 10 influenza virus-negative samples. Thus, Nanopore sequencing operated with 83% sensitivity (95% confidence interval [CI], 67 to 93%) and 100% specificity (95% CI, 69 to 100%) compared to the current diagnostic standard. Coverage of full-length virus was dependent on sample composition, being negatively influenced by increased host and bacterial reads. However, at high influenza virus titers, we were able to reconstruct >99% complete sequences for all eight gene segments. We also detected a human coronavirus coinfection in one clinical sample. While further optimization is required to improve sensitivity, this approach shows promise for the Nanopore platform to be used in the diagnosis and genetic analysis of influenza virus and other respiratory viruses.

## Abstract (precis)

Influenza is a major global public health threat as a result of its highly pathogenic variants, large zoonotic reservoir, and pandemic potential. Metagenomic viral sequencing offers the potential for a diagnostic test for influenza virus which also provides insights on transmission, evolution, and drug resistance and simultaneously detects other viruses. We therefore set out to apply the Oxford Nanopore Technologies sequencing method to metagenomic sequencing of respiratory samples.

## INTRODUCTION

Influenza A virus is an RNA orthomyxovirus of approximately 13 kb in length, with an eight-segment genome. It is typically classified on the basis of hemagglutinin (HA) and neuraminidase (NA), of which there are 16 and 9 main variants, respectively (1). Genetic reassortment underpins the potential for transmission between different host species (2) and for the evolution of highly pathogenic variants (3–6), recognized in the WHO list of “ten threats to global health” (7). Seasonal influenza causes an estimated 650,000 deaths globally each year, and the H3N2 variant alone kills 35,000 people each year in the United States (1, 8). Certain groups are particularly at risk, including older adults, infants, young children, pregnant women, those with underlying lung disease, and the immunocompromised (9). The burden of disease disproportionately affects low-/middle-income settings (10). Influenza virus diagnostics and surveillance are fundamental to identify the emergence of novel strains, to improve the prediction of potential epidemics and pandemics (4, 8), and to inform vaccine strategy (11). Diagnostic data facilitate real-time surveillance, can underpin infection control interventions (12, 13), and can inform the prescription of neuraminidase inhibitors (NAI) (9).

Currently, most clinical diagnostic tests for influenza virus depend on detecting viral antigen or on PCR amplification of viral nucleic acid derived from respiratory samples (14). These two approaches offer trade-offs in benefits, as follows: antigen tests (including point-of-care tests [POCT]) are typically rapid but have low sensitivity (15–17), while PCR is more time-consuming but more sensitive (9). Irrespective of the test used, most clinical diagnostic facilities report a nonquantitative (binary) diagnostic result, and the data routinely generated for influenza diagnosis have limited capacity to inform insights into epidemiological linkage, vaccine efficacy, or antiviral susceptibility. On these grounds, there is an aspiration to generate new diagnostic tests that combine speed (incorporating the potential for POCT [18, 19]), sensitivity, detection of coinfection (20, 21), and generation of quantitative or semiquantitative data that can be used to identify drug resistance and reconstruct phylogeny to inform surveillance, public health strategy, and vaccine design.

The application of Oxford Nanopore Technologies (ONT) sequencing to generate full-length influenza virus sequences from clinical respiratory samples can address these challenges. ONT offers a “third-generation,” portable, real-time approach to generating long-read single-molecule sequence data, with demonstrated success across a range of viruses (20, 22–24). To date, Nanopore sequencing of influenza virus has been reported using high-titer virus from an *in vitro* culture system, producing full-length genome sequences through direct RNA sequencing (25), or using targeted enrichment by either hybridization of cDNA (26) or influenza virus-specific PCR amplification (27).

We therefore aimed to optimize a metagenomic protocol for detecting influenza viruses directly from clinical samples using Nanopore sequencing. We determine its sensitivity compared to that of existing diagnostic methods and its accuracy compared to short-read (Illumina) sequencing, using clinical samples from hospital patients during an influenza season and samples from a controlled laboratory infection in ferrets. Further optimization is required before the Nanopore method can be rolled out as a diagnostic test, but we highlight the potential impact of this technology in advancing molecular diagnostics for respiratory pathogens.

## MATERIALS AND METHODS

### Study cohort and sample collection.

**FIG 1:**
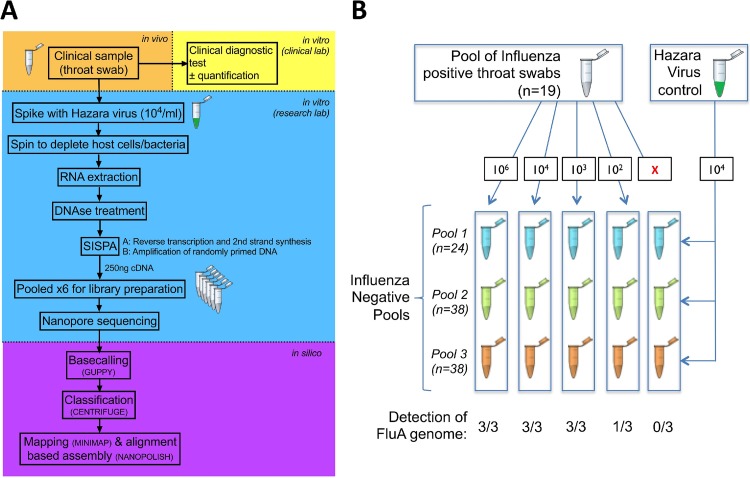
Schematic to show processing protocol through clinical and research pipelines for influenza diagnosis. (A) Clinical sample collection (orange), clinical diagnostic testing (yellow), sample processing and sequencing using Oxford Nanopore Technologies (blue), and processing of sequence data (purple). (B) Outline of pooled influenza virus-positive samples into an influenza virus-negative background to generate various titers of influenza virus (from 0 to 10^6^ genome copies/ml), undertaken in triplicate, and spiked with a standard titer of Hazara virus control at 10^4^ genome copies/ml. FluA, influenza A virus.

We collected respiratory samples from the clinical microbiology laboratory at Oxford University Hospitals NHS Foundation Trust, a large tertiary referral teaching hospital in Southeast England. We worked with anonymized residual material from throat and nose swabs generated as a result of routine clinical investigations between January and May 2018. Samples were collected using a sterile polyester swab inoculated into 1 to 3 ml of sterile viral transport medium (VTM), using a standard approach described on the CDC website (28). During the study, respiratory samples submitted to the clinical diagnostic laboratory were routinely tested by a PCR-based test using the GeneXpert assay (Cepheid) to detect influenza A and B viruses and respiratory syncytial virus (RSV). The workflow is shown in Fig. 1. Samples from patients in designated high-risk locations (hematology, oncology, and critical care) were tested using the BioFire FilmArray (bioMérieux) to detect an expanded panel of bacterial and viral pathogens. Quantitative data (cycle threshold [*C_T_*]) were generated by the GeneXpert assay, and we used the influenza virus *C_T_* value to estimate the viral titers in clinical samples. Using the GeneXpert assay, up to 40 PCR cycles are performed before a sample is called negative (i.e., positives have a *C_T_* value of <40). Quantification was not available for the BioFire results.

For methodological assessment, we focused on four categories of samples, as follows: positive pool, negative pools, individual positive samples, and individual negative samples. For the positive pool, we pooled 19 throat swab samples that had tested positive for influenza A virus in the clinical diagnostic laboratory to provide a large enough sample to assess reproducibility (Fig. 1B). For the negative pools, we generated three pools of throat swab samples that had tested negative for influenza virus (consisting of 24, 38, and 38 individual samples) (Fig. 1B). For the individual positive samples, we included 40 individual samples (35 throat swabs and 5 nasal swabs) that had tested positive for influenza A or B virus, selected to represent the widest range of GeneXpert assay *C_T_* values (13.5 to 39.3; valid test result range, 12 to 40). For the individual negative samples, we selected 10 individual throat swab samples that were influenza virus negative.

### Quantification of viral RNA in samples.

We quantified viral titers in Hazara virus stocks and pooled influenza A virus-positive throat swabs by quantitative reverse transcription-PCR (qRT-PCR), using previously described assays and standards (29, 30).

### Optimization of methods.

Prior to establishing the protocol detailed in full below, we assessed the impact of two possible optimization steps, centrifugation versus filtration and reduced time for cDNA synthesis. For centrifugation versus filtration, we investigated two approaches to deplete human/bacterial nucleic acid from our samples, i.e., filtration of the raw sample via a 0.4-μm filter (Sartorius) before further processing versus using a hard spin (16,000 × *g* for 2 min). cDNA libraries for this comparison were produced as described previously (20). For the reduced time for cDNA synthesis, to assess the possibility of time saving in the cDNA synthesis steps, we compared performance of the previously described protocol (20) to that of a modified version with two alterations, first using SuperScript IV (Thermo Fisher) in place of SuperScript III (Thermo Fisher) for reverse transcription, with the incubation time reduced from 60 min to 10 min at 42°C, and second, reducing the cDNA amplification PCR extension cycling time from 5 min to 2 min.

### Positive control.

Prior to nucleic acid extraction, each sample was spiked with Hazara virus virions to a final concentration of 10^4^ genome copies per ml as a positive internal control. This is an enveloped negative-stranded RNA virus (genus Orthonairovirus, order *Bunyavirales*) with a trisegmented genome of 11,980, 4,575, and 1,677 nucleotides in length (GenBank accession numbers KP406723 to KP406725). It is nonpathogenic in humans and would therefore not be anticipated to arise in any of our clinical samples. Cultured virions from an SW13 cell line were provided by the National Collection of Pathogenic Viruses (NCPV; catalog no. 0408084v).

### Nucleic acid extraction.

Samples were centrifuged at 16,000 × *g* for 2 min. The supernatant was eluted without disturbing the pelleted material and was used in nucleic acid extraction. Total nucleic acid was extracted from 100 μl of supernatant using the QIAamp viral RNA kit (Qiagen) eluting in 50 μl of H_2_O, followed by a DNase treatment with Turbo DNase (Thermo Fisher Scientific) at 37°C for 30 min. RNA was purified and concentrated to 6 μl using the RNA Clean & Concentrator-5 kit (Zymo Research), following the manufacturer’s instructions. Randomly amplified cDNA was prepared for each sample using a sequence-independent single-primer amplification (SISPA) approach, adapted from our previously described workflow (20), based on the round A/B methodology (23). For reverse transcription, 4 μl of RNA and 1 μl of primer A (5′-GTTTCCCACTGGAGGATA-N9-3′, 40 pmol/μl) (23) were mixed and incubated for 5 min at 65°C and then cooled to room temperature. First-strand synthesis was performed by the addition of 2 μl SuperScript IV first-strand buffer, 1 μl of 12.5 mM dinucleoside triphosphates (dNTPs), 0.5 μl of 0.1 M dithiothreitol (DTT), 1 μl H_2_O, and 0.5 μl SuperScript IV (Thermo Fisher) before incubation for 10 min at 42°C. Second-strand synthesis was performed by the addition of 1 μl Sequenase buffer, 3.85 μl H_2_O, and 0.15 μl Sequenase (Affymetrix) prior to incubation for 8 min at 37°C, followed by the addition of 0.45 μl Sequenase dilution buffer and 0.15 μl Sequenase and a further incubation at 37°C for 8 min. Amplification of cDNA was performed in triplicate using 5 μl of the reaction mixture as input to a 50-μl AccuTaq LA (Sigma) reaction mixture, according to the manufacturer’s instructions, using 1 μl primer B (5′-GTTTCCCACTGGAGGATA-3′) (23), with PCR cycling conditions of 98°C for 30 s, 30 cycles of 94°C for 15 s, 50°C for 20 s, and 68°C for 2 min, followed by 68°C for 10 min. Amplified cDNA was pooled from the triplicate reaction mixtures, purified using a 1:1 ratio of AMPure XP beads (Beckman Coulter, Brea, CA), and quantified using a Qubit high-sensitivity double-stranded DNA (dsDNA) kit (Thermo Fisher), both according to the manufacturers’ instructions.

### Nanopore library preparation and sequencing.

Multiplex sequencing libraries were prepared using 250 ng of cDNA from up to 12 samples as input to the SQK-LSK108 or SQK-LSK109 kit and barcoded individually using the EXP-NBD103 Native barcodes (Oxford Nanopore Technologies) and a modified One-pot protocol (https://www.protocols.io/view/one-pot-ligation-protocol-for-oxford-nanopore-libr-k9acz2e). Libraries were sequenced on FLO-MIN106 flow cells on the MinION Mk1b or GridION device (Oxford Nanopore Technologies), with sequencing proceeding for 48 h. Samples were batched according to the GeneXpert *C_T_* value (see File S1 in the supplemental material).

### Illumina methods.

Nextera XT V2 kit (Illumina) sequencing libraries were prepared using 1.5 ng of amplified cDNA, as per the manufacturer’s instructions, and sequenced on a 2 × 150-bp paired-end Illumina MiSeq run by the Genomics Services Development Unit of Public Health England.

### Bioinformatic analysis.

Nanopore reads were base called using Guppy (Oxford Nanopore Technologies, Oxford, UK). Output base called fastq files were demultiplexed using Porechop v0.2.3 (https://github.com/rrwick/Porechop). The reads were first taxonomically classified against the RefSeq database using Centrifuge v1.0.3 (31). The reads were then mapped against the reference sequence selected from the Centrifuge report using Minimap2 v2.9 (31, 32). A draft consensus sequence was generated by using a majority voting approach to determine the nucleotide at each position. The resulting draft consensus sequences were subjected to a BLAST search against an influenza virus sequence database that included >2,000 H1N1 and H3N2 seasonal influenza virus sequences between 2018 and 2019 and were downloaded from the Influenza Research Database (33). The reads were again mapped against the reference sequence using Minimap2 v2.9, and the number of mapped reads was calculated using SAMtools v1.5 (34) and Pysam (https://github.com/pysam-developers/pysam). The subtype of the influenza A virus derived from each clinical sample was determined by the subtypes of the HA and NA reference sequences. A consensus sequence was built using Nanopolish v0.11.0 (35, 36) and the margin_cons.py script (37) (https://github.com/zibraproject/zika-pipeline). For the Illumina data, reads were quality trimmed to a minimum score of Q30 across the read with Trimmomatic (38). BWA-MEM v0.7.15 (39) was used to align the reads to reference genomes using MEM defaults. SAMtools v1.4 was used to compute the percent reads mapped and coverage depth (34). Mapping consensuses for Illumina sequencing were generated using QuasiBam (40). Maximum likelihood phylogeny was generated for the HA gene segment using RAxML v8.2.10 (41), in which a general time-reversible model of nucleotide substitution and a gamma-distributed rate variation among sites were applied. Sequence alignments were performed by using MUSCLE v3.8 (42).

### Ferret study.

We applied our sequencing approach to residual samples collected in a previous time course experiment undertaken in a controlled laboratory environment (43). We tested ferret nasal saline wash samples from three independent animals over an 8-day time course, from 3 days prior to first exposure with influenza H1N1pdm09 virus and at days 1, 2, 3, and 5 postinfection. Sampling and plaque assays of the viral titer were described previously (43).

### Ethics approval.

The study of anonymized discarded clinical samples was approved by the London-Queen Square Research Ethics Committee (17/LO/1420). Ferret samples were residual samples from an existing study (43) for which the project license was reviewed by the local Animal Welfare and Ethics Review Board of Public Health England (Porton) and subsequently granted by the Home Office.

### Data availability.

Following the removal of human reads, our sequence data have been uploaded to the European Bioinformatics Institute (https://www.ebi.ac.uk/) under BioProject number PRJEB32861.

## RESULTS

### Method optimization to increase the proportion of viral reads derived from throat swabs.

Our method protocol is shown in Fig. 1A. We first sequenced five influenza A virus-positive and five influenza virus-negative throat swabs, each spiked with Hazara virus control at 10^4^ genome copies/ml. Using a sequence-independent single-primer amplification (SISPA) approach (20), followed by Nanopore sequencing, we produced metagenomic data dominated by reads that were bacterial in origin, with extremely few viral reads detected. Passing the sample through a 0.4-μm filter prior to nucleic acid extraction increased the detection of viral reads by several orders of magnitude (Fig. S1). Filtration is relatively expensive, so we also assessed the alternative approach of adding a rapid-centrifugation step to pellet bacterial and human cells, followed by nucleic acid extraction from the supernatant. We used a pooled set of influenza A virus-positive samples (concentration, 10^6^ genome copies/ml) to provide a large enough sample to assess reproducibility, with the Hazara virus control spiked in at 10^4^ genome copies/ml. Enrichment for influenza virus and Hazara virus was similar for filtration versus centrifugation, based on read mapping to the viral genome (Fig. S2). As centrifugation is simpler and less expensive, we selected this approach for all further testing.

### Method optimization to reduce time for cDNA synthesis.

Synthesis of tagged randomly primed cDNA and its subsequent amplification via SISPA (20) required lengthy reverse transcription and PCR steps (1 h and 3 h 45 min), respectively. Optimizing these stages upgraded the reverse transcriptase from SuperScript III to SuperScript IV (Thermo Fisher), reduced the incubation time to 10 min (processing time reduction, 50 min), and reduced the PCR extension time within each cycle from 5 min to 2 min (1 h 30 min processing time reduction). Comparing this final method with our original protocol, using triplicate extractions from the pooled set of influenza A virus-positive samples demonstrated no significant loss in performance in the more rapid protocol (Fig. S3), and we adopted this approach as our routine protocol, giving a wet-lab processing time of ∼8 h.

### Consistent retrieval of Hazara virus by Nanopore sequencing.

**FIG 2:**
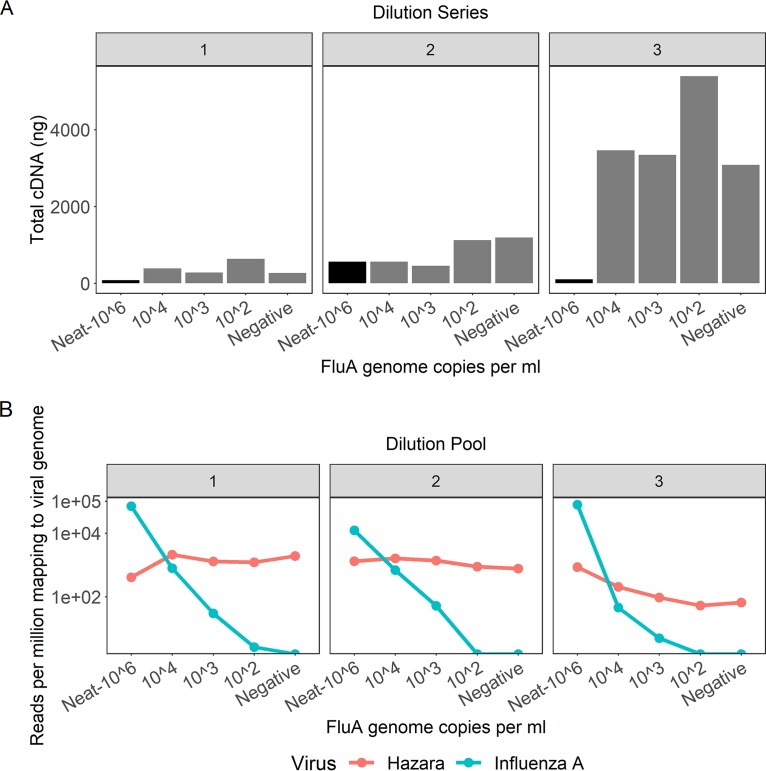
Characteristics of three pools of influenza virus-negative throat swabs and Nanopore sequence results following spiking with influenza A virus. (A) Total concentration of cDNA produced per pooled sample following amplification by the SISPA reaction, grouped by dilution series. The 10^6^ genome copies/ml sample in each pool is the original, undiluted material, represented by the black bars. Samples diluted to influenza virus titers of 10^4^, 10^3^, and 10^2^ contain more cDNA due to higher background material (bacterial/human) present in the diluent. Dilution series 1 and 2 contain comparable amounts of background material; dilution series 3 contains substantially more background. (B) Viral reads generated by Nanopore sequencing of samples with different titers of influenza A virus and a consistent titer of Hazara virus (10^4^ genome copies/ml). Graphs show reads per million of total reads mapping to influenza A or Hazara virus genomes, across the three individual dilution series. Note the logarithmic scale on the *y* axis.

Starting with an influenza A virus-positive sample pool (10^6^ genome copies/ml), we made three volumetric dilution series using three independent influenza virus-negative pools (Fig. 1B). The total quantity of cDNA after preparation for sequencing was consistently higher in all samples using negative pool 3 as the diluent (Fig. 2A), indicating the presence of a higher concentration of nonviral RNA within pool 3. This is likely due to host cell lysis or higher bacterial presence and demonstrates the variable nature of throat swab samples.

We consistently retrieved Hazara virus reads from all three dilution series by Nanopore sequencing, independently of influenza virus titer in the sample (Fig. 2B). Sequencing from dilution series 1 and 2 gave a consistent proportion of total reads mapping to the Hazara virus genome, across dilutions and between the first two pools, with mean ± standard deviation values per pool of 1.4 × 10^3^ ± 660 reads per million (RPM) of total reads and 1.2 × 10^3^ ± 350 RPM, respectively. The pool 3 dilution series generated 260 ± 340 RPM Hazara virus reads across samples and showed a decreasing trend associated with increased dilution factor as increasingly more nonviral RNA was introduced from this high-background pool.

### Limit of influenza virus detection by Nanopore sequencing from pooled samples.

Nanopore sequencing of the triplicate SISPA preparations of the influenza A virus-positive pool produced mean ± standard deviation of 5.3 × 10^4^ ± 3.6 × 10^4^ RPM mapping to the influenza A virus genome (Fig. 2B). Across the dilution series, the proportion of influenza virus reads was strongly associated with influenza virus titer (*P* value = 4.7 × 10^−5^) but was also influenced by which negative pool was used for dilution, consistent with the pattern observed for the Hazara virus control. Sequencing the negative controls (pools with no influenza virus spike) generated no reads mapping to influenza virus. At influenza virus titers of <10^3^ copies/ml, influenza virus reads were inconsistently detected across the samples (Fig. 2B), suggesting that the limit of detection is between 10^2^ and 10^3^ influenza virus copies/ml.

### Retrieval and reconstruction of complete influenza virus genomes from pooled/spiked samples.

**FIG 3:**
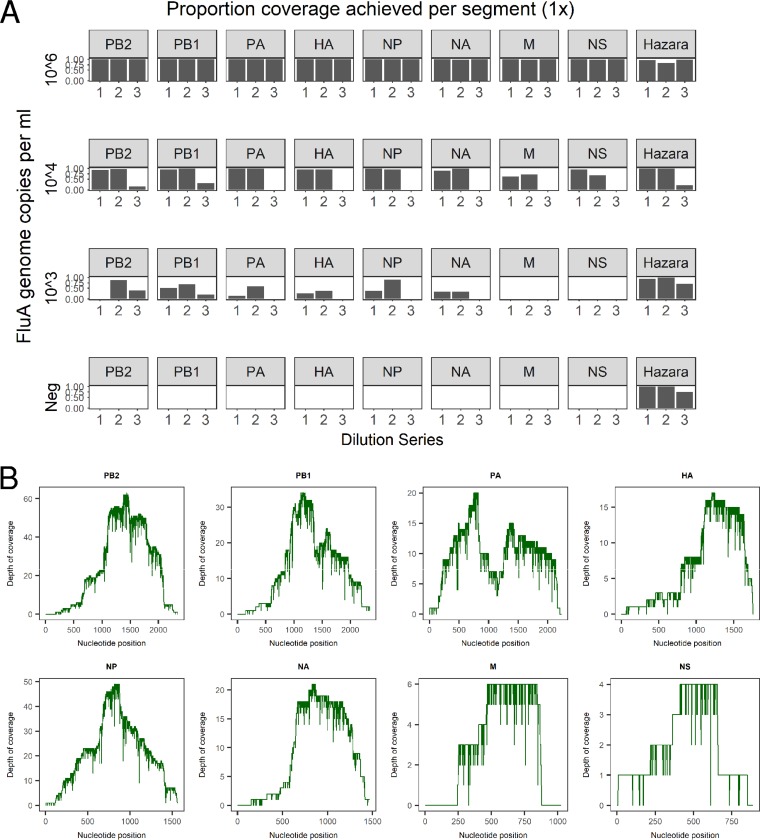
Coverage of influenza virus and Hazara virus genome segments achieved by Nanopore sequencing from pooled samples (A) Data from three dilution series of pooled influenza virus-positive samples, diluted with three separate negative-sample pools to generate different titers of influenza virus. Each individual dilution was spiked with Hazara virus at 10^4^ genome copies/ml. The proportion of genome covered at 1× depth is shown for each of the eight influenza virus genome segments (encoding PB2 [polymerase subunit 2], PB1 [polymerase subunit 1], PA [polymerase acidic protein], HA [hemagglutinin], NP [nucleocapsid protein], NA [neuraminidase], M [matrix protein], and NS [nonstructural protein]) across the three dilution series. For simplicity, the coverage of the Hazara virus genome is plotted as the total of all three genome segments. (B) Representative coverage plots of influenza A virus genome segments from the dilution series 1 sample at 10^4^ influenza virus copies per ml.

For the Hazara virus control (10^4^ genome copies/ml spike), genome coverage was 81.4 to 99.4% (at 1× depth) for pools 1 and 2. Coverage in the high-background pool 3 was more varied (21.5 to 96.5%; Fig. 3A). Influenza A virus genome coverage at 10^6^ copies/ml was ≥99.3% for each segment in all samples (Fig. 3A). At 10^4^ genome copies/ml of influenza virus, a mean 1× coverage per segment was 90.3% for pools 1 and 2 but was substantially reduced in the high-background pool 3 to 5.7% (Fig. 3A). At influenza virus titers of <10^4^ copies/ml, coverage was highly varied across genome segments. However, when present at 10^3^ copies/ml, 2/3 pools had sufficient data for correct subtyping as H3N2 (Table 1).

**TABLE 1:** Summary of results from Nanopore sequencing based on pooled samples with various titers of influenza A virus and a consistent titer of Hazara virus control^*a*^

Influenza A virus titer (genome copies/ml)	Dilution pool no.	Total no. of reads	No. of influenza A virus reads (reads per million)	Influenza A virus subtyping^*b*^	No. of Hazara virus reads (reads per million)
10^6^	1	473,718	33,103 (6.9 × 10^4^)	H3N2	527 (1.1 × 10^3^)
	2	572,106	6,957 (1.2 × 10^4^)	H3N2	102 (178)
	3	526,852	41,196 (7.8 × 10^4^)	H3N2	534 (1.0 × 10^3^)
10^4^	1	354,163	280 (791)	H3N2	738 (2.1 × 10^3^)
	2	433,033	299 (690)	H3N2	691 (1.6 × 10^3^)
	3	43,512	2 (46)	Not possible	9 (207)
10^3^	1	231,929	7 (30)	H3N2	298 (1.3 × 10^3^)
	2	461,281	24 (52)	H3N2	638 (1.4 × 10^3^)
	3	397,672	2 (5)	Not possible	38 (96)
10^2^	1	375,183	1 (3)	Not possible	453 (1.2 × 10^3^)
	2	671,133	0 (0)	Not possible	598 (891)
	3	37,897	0 (0)	Not possible	2 (53)
Negative	1	903,430	0 (0)	NA	1,731 (1.9 × 10^3^)
	2	900,471	0 (0)	NA	692 (768)
	3	818,549	0 (0)	NA	54 (66)

a Each dilution is undertaken in triplicate (shown as 3 dilution pools).

b NA, not applicable.

### Influenza virus detection from individual clinical samples.

Having demonstrated our ability to retrieve influenza virus sequences from pooled influenza virus-positive material diluted with negative samples, we next applied our methods to individual anonymized clinical samples, with 40 samples testing influenza virus positive and 10 samples testing influenza virus negative in the clinical diagnostic laboratory. Data yield varied between flow cells (range, 2.5 × 10^6^ to 13.2 × 10^6^ reads from up to 12 multiplexed samples). Within flow cells, barcode performance was inconsistent when using a stringent, dual-barcode, demultiplexing method (21). From each clinical sample, the range of total reads generated was 1.0 × 10^5^ to 2.4 × 10^6^ (median, 3.8 × 10^5^ reads) (Table S1).

**FIG 4:**
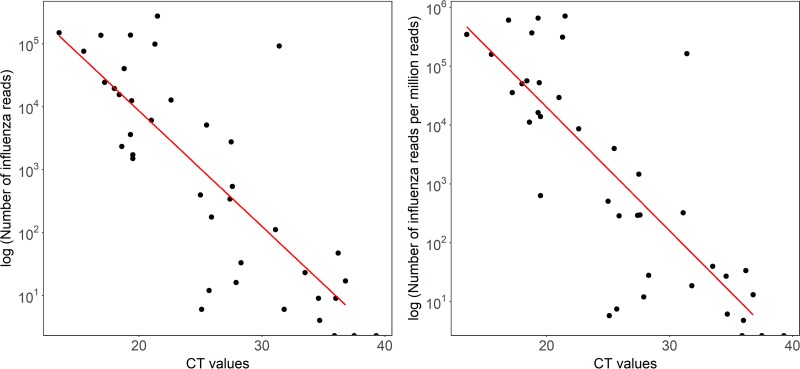
Total and proportion of influenza virus reads derived by Nanopore sequencing of individual samples across a range of *C_T_* values. *C_T_* values were derived by testing using the GeneXpert (Cepheid) assay in a clinical diagnostic laboratory. Left, correlation between *C_T_* value and total number of influenza virus reads generated. *R*^2^ = 0.604, *P* = 2.47e−08. Right, correlation between *C_T_* value and number of influenza virus reads per million reads. *R*^2^ = 0.623, *P* = 1.07e−08.

Reads mapping to either the influenza A or B virus genome were present in all 27 samples with a *C_T_* of <30 (range, 6 to 274,955 reads). At a *C_T_* of >30, 6/13 samples generated influenza virus reads (range, 6 to 92,057 reads) (difference between sensitivity at a *C_T_* threshold of 30, *P* < 0.0001; Fig. 4). The highest *C_T_* value at which any influenza virus reads were detected was 36.8 (sample 37; 17 reads of influenza A virus). No reads classified as influenza virus were obtained from sequencing the 10 GeneXpert assay-negative samples (Table S1). Based on this small data set, sensitivity is 83% and specificity is 100% (95% CI, 67 to 93% and 69 to 100%, respectively). There was a strong correlation between *C_T_* value and both the reads per sample classified as influenza virus (*R*^2^ = 0.60) and the number of influenza virus reads per million reads (*R*^2^ = 0.62) (Fig. 4). The consensus genome sequences generated (at 10× minimum depth) covered over 90% of the influenza virus genome for 17 samples, with another two generating over 80% coverage. The highest *C_T_* value of a sample from which >90% of an influenza virus genome sequence was generated was 27.5 (Fig. S4).

### Detection of Hazara virus internal control.

Detection of the control virus (Hazara virus at 10^4^ genome copies/ml) was highly varied, demonstrating that levels of background nontarget RNA are a major source of intersample variation. The number of Hazara virus reads per sample ranged from 0 to 13.5 × 10^3^ (0 to 3.5 × 10^4^ RPM), with a median of 70 reads (160 RPM) and mean of 706 reads (1.7 × 10^3^ RPM) (Table S1). Four (8%) of 50 samples generated no detectable Hazara virus reads, two with high numbers of influenza virus reads (for sample 1, *C_T_* of 13.5 and 1.5 × 10^5^ influenza B virus reads, and for sample 6, *C_T_* of 18.4 and 1.5 × 10^4^ influenza A virus reads) acting to dilute the control signal. The other two samples contained no detectable influenza virus reads (for sample 34, *C_T_* of 35.9, and for sample 46, influenza virus negative). The lack of control detection therefore indicates a loss of assay sensitivity due to high levels of background nucleic acid present in some samples.

### Comparison of Nanopore and Illumina sequencing.

We selected a subset of 15 samples from across the viral titer range and resequenced on an Illumina MiSeq platform. The proportions of reads generated that mapped to the influenza virus genome were similar between the two sequencing technologies (Fig. S5). From 4 of the samples, nearly complete genomes were obtained. A comparison of consensus sequences derived from Nanopore and Illumina sequencing showed 100% concordance, except one sample that showed 7 nucleotide differences (identity, 99.94%) (Table S2).

### Influenza virus phylogeny.

**FIG 5:**
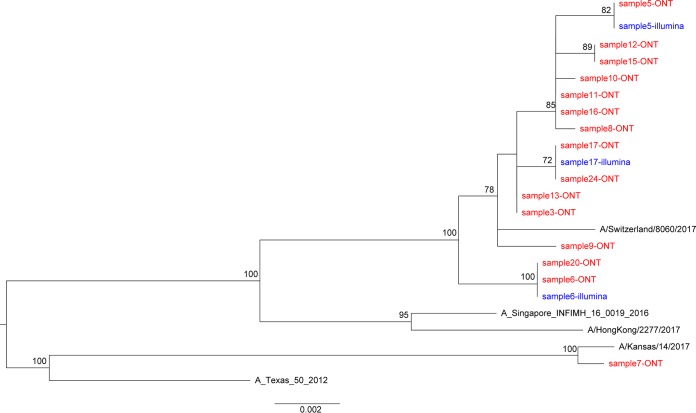
Phylogenetic trees of consensus influenza virus HA gene derived by Nanopore and Illumina sequencing. A maximum likelihood tree was generated using 500 bootstrap replicates in the RAxML v8.2.10 software. Bootstrap values of >70 are shown. The scale bar shows the substitutions per site. Red and blue indicate sequences derived from Oxford Nanopore Technology (ONT) and Illumina sequencing, respectively. Reference sequences are shown in black.

We reconstructed the phylogeny using consensus sequences for the HA gene (Fig. 5). This demonstrates closely related sequences, as expected within one geographic setting in a single influenza season.

### Detection of other RNA viruses in clinical samples.

Within the 50 clinical samples sequenced, we found limited evidence for the presence of other RNA viruses. Sample 6 produced 109 reads mapping to human coronavirus in addition to >1.5 × 10^4^ influenza A virus reads, suggesting coinfection. We also derived >4.0 × 10^4^ reads from human metapneumovirus from an influenza virus-negative sample, providing a nearly complete genome (99.8% coverage) from one sample (Fig. S1, sample I), further detailed previously (44).

### Animal time course study.

**FIG 6:**
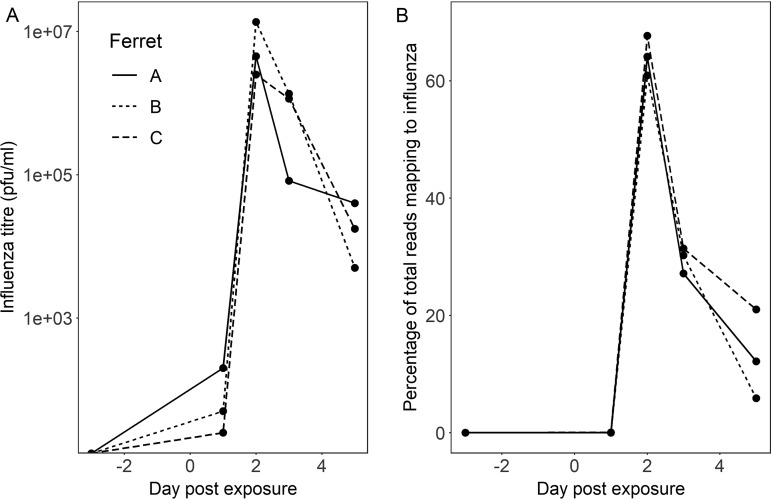
Time course experiment showing influenza A virus infection in three laboratory ferrets. Infection was introduced at day 0. Samples were collected 3 days prior to infection and at days 1, 3 and 5 postinfection. (A and B) Influenza virus titer (log scale) (A) and proportion of total Nanopore reads (linear scale) (B) mapping to influenza A virus from metagenomic sequencing of ferret nasal washes taken before and after influenza virus challenge.

Finally, we used samples collected from a previous animal experiment (43) to test the reproducibility of our methods across a time course model of influenza A virus infection (three ferrets swabbed preinfection [day −3] and then sampled at days 1, 2, 3, and 5 following laboratory infection with influenza A virus). The proportion of viral reads present at each time point was highly congruent with viral titer (titer is shown in Fig. 6A and sequencing reads in Fig. 6B). We generated consensus genome sequences from Nanopore data at days 2, 3, and 5 postinfection; these were 100% concordant with Illumina-derived consensus sequences from the same cDNA (Table S2).

## DISCUSSION

To our knowledge, this is the first report of successfully applying metagenomic Nanopore sequencing directly to respiratory samples to detect influenza virus and generate influenza virus sequences. The approach demonstrates excellent specificity. Sensitivity varies by viral titer but is comparable to that of existing laboratory diagnostic tests for *C_T_* values of <30. Our optimized protocol depletes human and bacterial nucleic acids and reduces the time from sample to sequence. This method has the potential to be further optimized and validated to improve sensitivity for influenza virus, identify other RNA viruses, detect drug resistance mutations, and provide insights into quasispecies diversity (45, 46). At a population level, these sequence-based diagnostic data can, in addition, provide phyloepidemiological reconstruction, insights into transmission events, the potential to estimate vaccine efficacy (47), and approaches for public health intervention (48).

Whole-genome viral sequencing, coupled with phylogenetic analysis and appropriate clinical metadata, can contribute to the accurate tracking of outbreaks across time and space (49). The metagenomic method employed here produced >90% complete genomes for 17/27 samples with a *C_T_* value of ≤30 (Fig. S4), demonstrating the ability of metagenomics to produce sufficient data for influenza virus diagnostics and genome characterization, while also detecting and sequencing other common RNA viruses.

Despite time reductions in wet-laboratory processing, this method requires further modification to simplify and accelerate the protocol if it is to become viable as a near-to-patient test. High error rates are a recognized concern in Nanopore sequence data, and cross-barcode contamination can create challenges when low- and high-titer samples are batched (21). To avoid these problems, we batched samples according to *C_T_* value and applied stringent barcode demultiplexing criteria; however, this reduces the total data available for analysis, typically by ∼50% but with variation between sequencing runs (21). For future primary diagnostic use, it would be preferable to sequence samples individually using a lower-throughput flow cell, e.g., ONT Flongle (each paired with a negative-extraction-control sample, for which a prior spike with Hazara virus, using the same methods described here, would remain appropriate). Careful optimization of laboratory and bioinformatic methods is required to resolve individual sequence polymorphisms, particularly for drug resistance alleles.

Infectious Diseases Society of America (IDSA) guidelines (9) recommend nasal/nasopharyngeal specimens for influenza diagnosis, but throat swabs are easier to collect in clinical practice and therefore account for the majority of diagnostic samples processed by our clinical microbiology laboratory. Further work is needed to investigate the sensitivity and specificity of our protocol for a wider array of respiratory sample types (also including bronchoalveolar lavage fluid, sputum, and saliva), which may contain different degrees of contaminating bacterial and/or human reads. Loss of assay sensitivity due to the presence of high-level background DNA from either the host or bacterial origin is a fundamental issue for metagenomic approaches, even in cell-free sample types such as cerebrospinal fluid (50). This challenge is exacerbated in throat swabs, as seen in our data. Our use of Hazara virus as an internal positive control allows us to identify those samples in which sensitivity has dropped to <10^4^ viral genome copies per ml. In our test set, 8% of samples showed insufficient sensitivity for Hazara virus; however, half of these contained a high titer of influenza virus, so only 4% were true sensitivity failures. This figure is in line with the reported 6% failure rate due to high background for RNA virus detection from a clinically validated metagenomic sequencing assay for pathogen detection in cerebrospinal fluid (50).

At the higher *C_T_* values in our clinical samples (*C_T_*, 30 to 40), the sensitivity of Nanopore sequencing was reduced compared to that of the current PCR-based test (GeneXpert assay; Cepheid). Further optimization will be required to maximize the diagnostic yield from this group of samples without sacrificing specificity.

The correlation between *C_T_* value and Nanopore reads confirms semiquantitative output. Using samples from the ferret influenza virus model, collected under standardized laboratory conditions, we demonstrated excellent reproducibility of viral read proportions at a given viral titer across biological replicates. However, we observed heterogeneity in output between clinical samples as well as between Nanopore flow cells, suggesting that the current platform is not yet sufficiently reliable for reproducibly generating quantitative data. In addition, the detection of positive controls can be impaired in high-background samples.

Future application of this method will involve real-time laboratory testing of respiratory samples, running the platform head to head with existing clinical diagnostics to further assess sensitivity and specificity, and using influenza virus sequence data to investigate transmission events. Identifying instances of nosocomial transmission may shed light on health care-acquired infection, thus helping to improve infection control practice. Assessment of diversity within deep-sequence data sets provides an opportunity to investigate the relationship between within-host polymorphisms and clinical outcomes. Long-read sequences confer the potential advantage of identifying viral haplotypes and ascertaining the extent to which significant polymorphisms are transmitted together or independently (24). We have shown that the method is robust for the identification of commonly circulating influenza virus strains in human populations, but further investigation is required to ascertain the extent to which it performs reliably in other (avian and animal) strains.

### Comparison with existing/alternative approaches.

The current standard assay for influenza diagnosis employed within the large tertiary referral teaching hospital in which this study was performed is the GeneXpert assay (Cepheid), which detects influenza A and B viruses and respiratory syncytial virus (RSV). Wider testing is performed on a subset of samples using the BioFire FilmArray respiratory panel (bioMérieux), targeted at 20 common respiratory pathogens (17 viruses and 3 bacteria). These assays have the advantages over a metagenomic approach of higher sensitivity, shorter handling times, simpler laboratory workflow, and very rapid time to result (30 and 65 min, respectively). Compared to a metagenomic approach, their limitations are that no sequence data informative for molecular epidemiology and drug resistance typing are generated for the target pathogens, and that the assay will only detect the small number of pathogens targeted. Periodic refinement of such assays is required in the event of newly emergent pathogens or diverse strains of established pathogens leading to assay escape. This is not an issue affecting metagenomic sequencing, which has the ability to detect all RNA viruses in a sequence-independent manner. At the time of undertaking this laboratory work, the materials costs of the metagenomic sequencing were ∼£140 per sample when multiplexing six samples per flow cell and purchasing 48 flow cells together. The cost of the current influenza A/B and RSV GeneXpert test is ∼£56, and the BioFire RP panel costs ∼£139 per sample. The existing tests have the significant advantage of short handling times and simple processing, whereas the metagenomic sequencing requires ∼8 h and skilled laboratory staff.

Alternative approaches to generate sequence data include amplicon-based sequencing of the influenza virus genome (51, 52). However, this approach detects only the target pathogen, requiring multiple assays or more complex multiplex primer schemes to add targets and capture diverse strains of the original target. The use of short-read Illumina sequencing instead of Nanopore sequencing for metagenomic sequencing of influenza virus (53) provides the current gold standard of sequence quality and some potential cost savings per base of sequence generated. However, our data show that at a relatively modest minimum coverage depth of 10×, Nanopore-generated consensus viral genome sequences are 99.95 to 100% identical to Illumina sequences. Of the 13 samples compared, 12 samples were 100% concordant. The few bases that differed between the technologies in a single sample appear to be clear in each case and a genuine disagreement between the long- and short-read approaches, rather than simply being due to the higher per-base error rate of Nanopore *per se*. Larger and more diverse data sets will be required to set more rigorous thresholds for base calling. Currently, it is wise to bear such potential issues in mind when comparing genome sequences generated by different technological platforms.

The per-read differences in base accuracy are also compensated for by the increased read length, providing further confidence in the case of individual read taxonomic assignment. Consideration of relative costs must also take into account other cost-saving attributes. These include the substantially lower infrastructure and startup costs of Nanopore sequencing, the unique ability to interrogate sequence data as they are generated in real time, and the potential for the portable MinION device to be utilized near to patient, potentially decreasing turnaround time, particularly for high-virus-load samples which may be identified within minutes. The throughput of the Nanopore flow cells allows for a small number of samples to be run immediately rather than requiring samples to be batched to reach a number sufficient to cost efficiently run a greater-throughput short-read sequencing device. In the future, the use of an even lower-throughput flow cell with the ONT Flongle adaptor may allow individual samples to be run per cell, offering quicker turnaround per sample and minimizing cross-sample contamination. A summary table comparing the different approaches is included in supplemental material (Table S3).

### Limitations of the method.

The current limitations of metagenomic methods are their sensitivity in the context of low-pathogen-titer samples. PCR-based methods measure the absolute count of viral genome copies present within a sample. Metagenomic sequencing measures the proportion of total RNA that is viral. Metagenomic sequencing is therefore affected by the level of nontarget RNA within a given sample, whereas PCR is not. As demonstrated here (Fig. 2b and Table 1), detection of as little as 10^2^ genome copies per ml is possible from throat swab samples (a level comparable with PCR-based methods), but variation in the level of background nucleic acids between individual samples makes detection at this level inconsistent. Further development of methods to deplete host and bacterial RNA within the samples is required to improve the performance of the assay at *C_T_* values of >30. Enrichment of pathogen sequences within libraries through either target capture or amplification is also an effective method to reduce the limit of target detection (54, 55) but requires the same *a priori* knowledge of both which pathogens are to be targeted and the full range of circulating viral diversity as other targeted methods discussed above, albeit with increased tolerance for diversity over PCR-based methods. A further limitation compared to alternative sequencing technologies is the lack of confidence in determining the presence of minority variants due to the limited per-read accuracy, although we expect this to be addressed in future iterations of the ONT sequencing.

In summary, while substantial further work is needed, our methods show promise for generating influenza virus sequences directly from respiratory samples. The “pathogen-agnostic” metagenomic sequencing approach offers an opportunity for simultaneous testing for a wide range of potential pathogens, providing a faster route to optimum treatment and contributing to antimicrobial stewardship. Longer term, this approach has promise as a routine laboratory test, providing data to inform treatment, vaccine design and deployment, infection control policies, and surveillance.

## Supplementary Material

Supplemental file 1
