# Supplementary material for: Metagenomic Nanopore Sequencing of Influenza Virus Direct from Clinical Respiratory Samples
Source: J Clin Microbiol. 2019 Dec 23;58(1):e00963-19. doi: 10.1128/JCM.00963-19 (PMC6935926; doi:10.1128/JCM.00963-19)
Supplement: Supplemental file 1 [file JCM.00963-19-s0001.pdf]

Supplementary material

Figure S1 Comparison of viral read detection in throat swab samples with and without a filtration step. Five influenza A positive throat swab samples (A-E) and five negative samples (F-J) were spiked with Hazara virus as positive control at 10^4 genome copies/ml and sequenced with and without filtration via a 0.4µm filter prior to RNA extraction. Reads mapping to influenza or Hazara are shown for each sample.

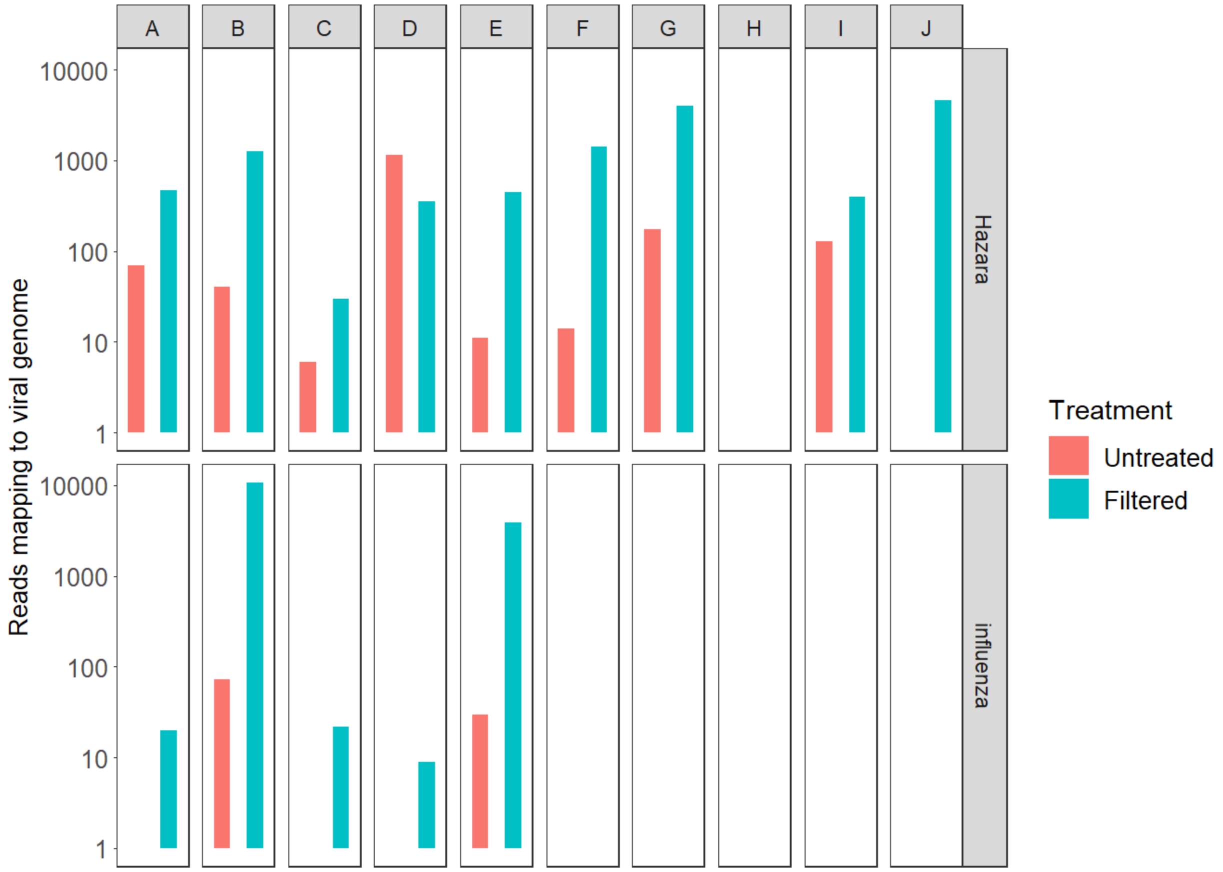

Figure S2 Comparison of Filtration and Centrifugation as Pre-extraction treatments to improve viral detection. Using a pool of 19 influenza A positive samples as input, triplicate extractions utilising either supernatant separation by centrifugation at 16,000 xg for 2 min, or filtration via a 0.4µm filter, were processed and all samples sequenced on a single flow cell. Mean total read numbers for each virus are plotted for each treatment, error bars indicate standard deviation.

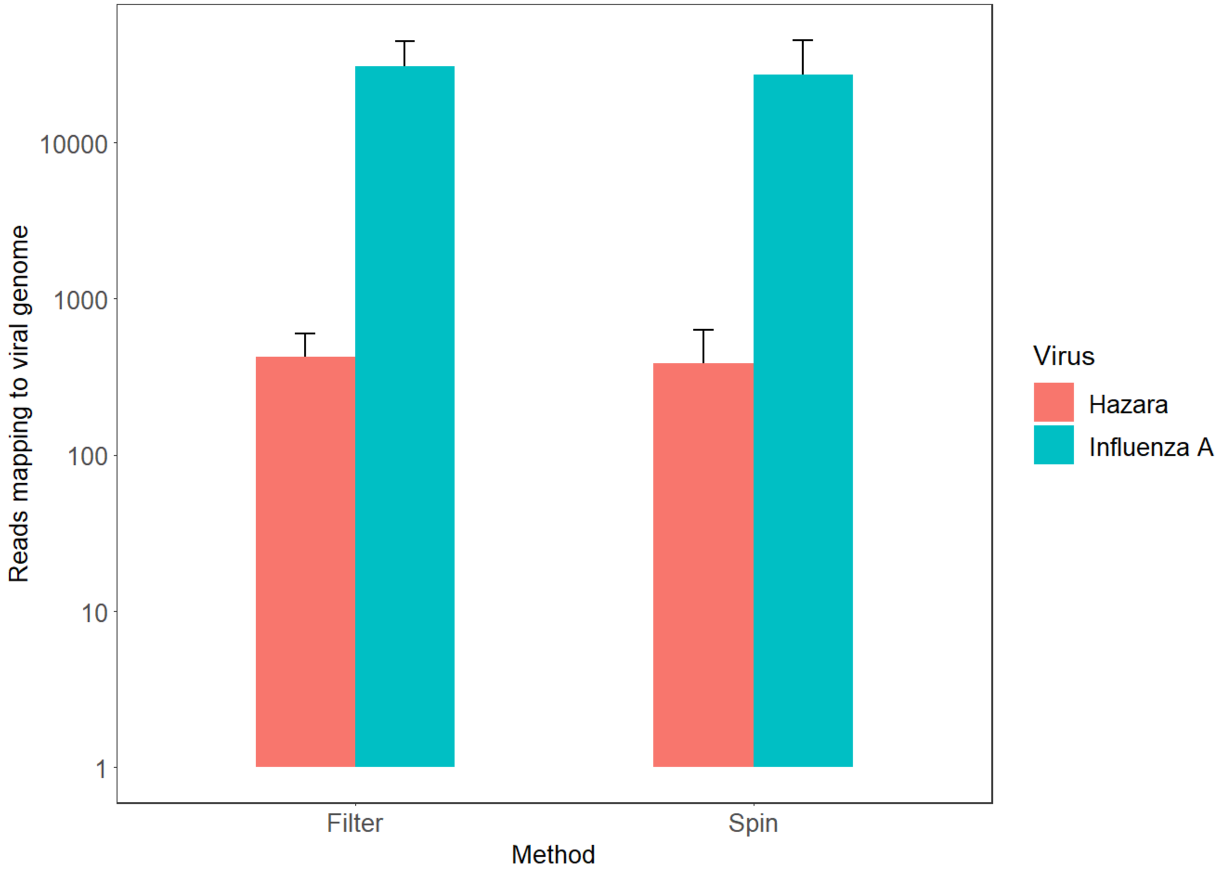

Figure S3. Assessment of effect of original and reduced processing time method on metagenomic viral sequencing. Using a pool of influenza A positive samples as input, triplicate extractions were processed by either existing (standard) or reduced incubation times (rapid) method and all samples sequenced on a single flow cell. Mean total read numbers for each virus are plotted for each treatment, error bars indicate standard deviation.

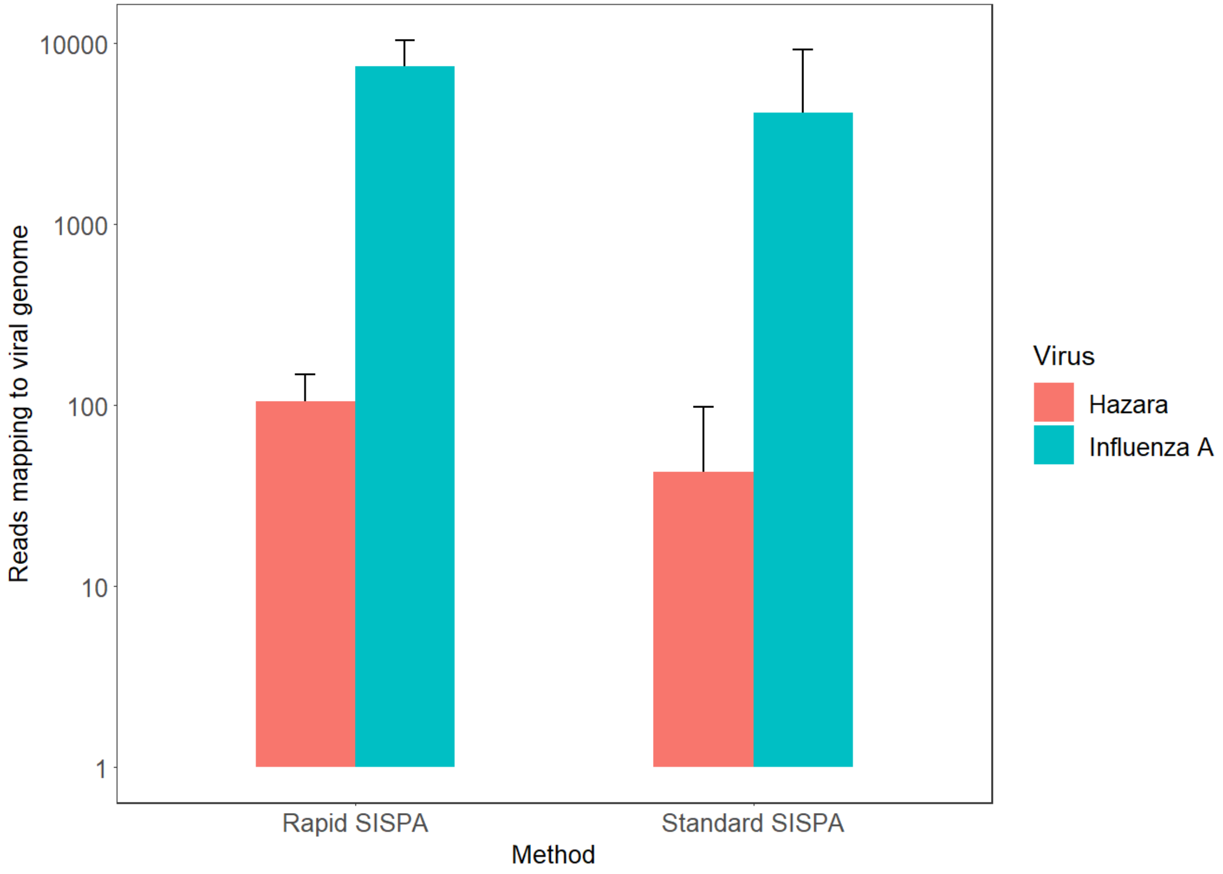

Figure S4. Coverage of influenza sequences recovered by nanopore metagenomic sequencing. Ct values were derived by testing using GeneXpert (Cepheid) in a clinical diagnostic laboratory. Genome coverage shown is the proportion of bases of the reference sequence that were called in the final consensus sequence for each sample.

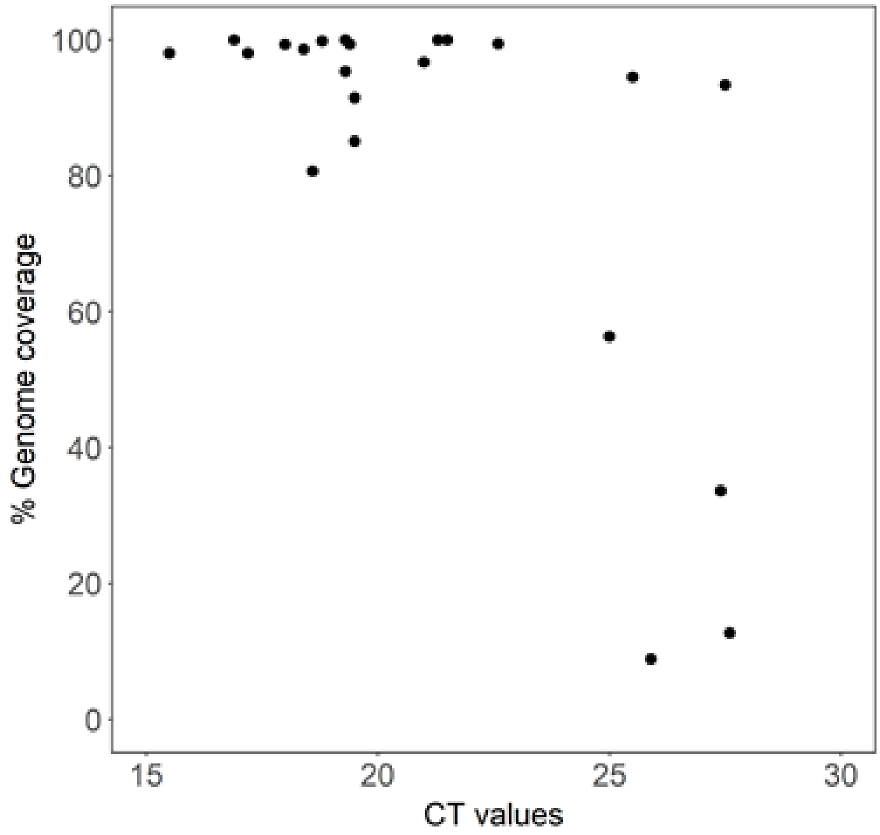

Figure S5. Comparison of proportions of influenza reads derived by Nanopore or Illumina sequencing for a subset of the individual samples across a range of Ct values. Ct values were derived by testing using GeneXpert (Cepheid) in a clinical diagnostic laboratory. Proportions are shown as number of influenza reads per million reads.

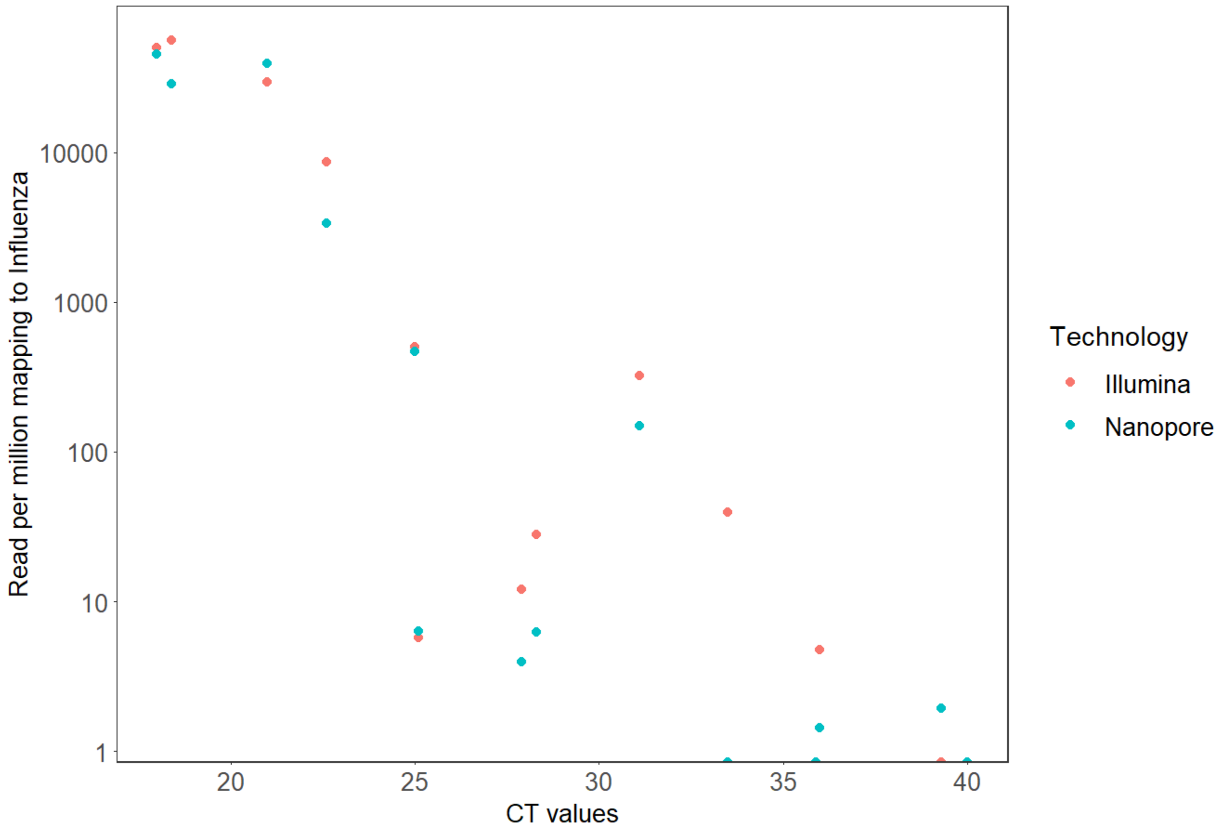

Table S1. Summary data for the 50 individual throat swab samples.
Table is available on Figshare DOI: 10.6084/m9.figshare.9772034

Table S2 Comparison of influenza consensus sequences derived from Nanopore and Illumina sequencing. This table lists the 4/15 human samples that gave near complete genome coverage, together with all 9 samples derived from the ferret study

Study ID	Cepheid Ct	ONT consensus coverage (%)	Illumina consensus coverage (%)	Nucleotide mismatch (identity %)
Sample 5	18	99	100	0 (100)
Sample 6	18.4	99	100	0 (100)
Sample 14	21	97	98	7 (99.94)
Sample 17	22.6	99	84	0 (100)
A d2	n/a	100	100	0 (100)
A d3	n/a	100	100	0 (100)
A d5	n/a	100	100	0 (100)
B d2	n/a	100	100	0 (100)
B d3	n/a	100	100	0 (100)
B d5	n/a	98	93	0 (100)
C d2	n/a	100	100	0 (100)
C d3	n/a	100	100	0 (100)
C d5	n/a	100	100	0 (100)

Note: “Sample” indicates clinical human sample;, A/B/C indicate samples from ferrets at indicated days post infection. n/a=not available

Table S3. Comparison of influenza diagnostics approaches .

	GeneXpert FluA/B RSV	Biofire Respiratory Panel	Nanopore Metagenomic sequencing	Nanopore targeted Flu sequencing	Illumina Metagenomic sequencing
**Reagent cost per test**	+	+++	+++	++	++
**Equipment/Start up cost**	+++	+++	+	+	+++
**Wet lab time**	+	+	+++	++	+++
**Sensitivity**	+++	+++	+	++	+
**Target range**	+ Flu A/B, RSV	++ 18 viruses 4 bacteria (latest RP2)	+++ All RNA viruses	+ Flu A/B	+++ All RNA viruses
**Advantages**	Rapid and highly sensitive for influenza and RSV. No batching required.	Rapid and highly sensitive for common pathogens. No batching required	Wide target range. Produces sequence data in real-time. Potential for near-patient testing. Small batch size. Accurate basecalling at consensus level.	Produces sequence data. Better sensitivity than pathogen agnostic methods.	Wide target range. Produces sequence data. Accuracy sufficient for minor variant analysis
**Disadvantages**	Extremely limited target range. No info beyond presence / absence of 3 viruses	Expensive / limited range of targets. No sequence info generated.	Requires expertise / slow / reduced sensitivity for influenza. Potential concerns about error rate in deep sequences.	Limited target range.	Large start-up investment, large batch sizes required for cost effective running
